# Birds in Anthropogenic Landscapes: The Responses of Ecological Groups to Forest Loss in the Brazilian Atlantic Forest

**DOI:** 10.1371/journal.pone.0128923

**Published:** 2015-06-17

**Authors:** José Carlos Morante-Filho, Deborah Faria, Eduardo Mariano-Neto, Jonathan Rhodes

**Affiliations:** 1 Applied Conservation Ecology Lab, Programa de Pós-graduação Ecologia e Conservação da Biodiversidade, Universidade Estadual de Santa Cruz, Rodovia Ilhéus-Itabuna, km16 /Salobrinho, Ilhéus, Bahia, Brazil; 2 Instituto de Biologia, Universidade Federal da Bahia, R. Barão Jeremoabo, Ondina, Salvador, Bahia, Brazil; 3 School of Geography, Planning and Environmental Management, The University of Queensland, St Lucia, Brisbane, Queensland, Australia; 4 ARC Centre of Excellence for Environmental Decisions, The University of Queensland, Brisbane, Queensland, Australia; The Australian National University, AUSTRALIA

## Abstract

Habitat loss is the dominant threat to biodiversity and ecosystem functioning in terrestrial environments. In this study, we used an *a priori* classification of bird species based on their dependence on native forest habitats (forest-specialist and habitat generalists) and specific food resources (frugivores and insectivores) to evaluate their responses to forest cover reduction in landscapes in the Brazilian Atlantic Forest. From the patch-landscapes approach, we delimited 40 forest sites, and quantified the percentage of native forest within a 2 km radius around the center of each site (from 6 - 85%). At each site, we sampled birds using the point-count method. We used a null model, a generalized linear model and a four-parameter logistic model to evaluate the relationship between richness and abundance of the bird groups and the native forest amount. A piecewise model was then used to determine the threshold value for bird groups that showed nonlinear responses. The richness and abundance of the bird community as a whole were not affected by changes in forest cover in this region. However, a decrease in forest cover had a negative effect on diversity of forest-specialist, frugivorous and insectivorous birds, and a positive effect on generalist birds. The species richness and abundance of all ecological groups were nonlinearly related to forest reduction and showed similar threshold values, i.e., there were abrupt changes in individuals and species numbers when forest amount was less than approximately 50%. Forest sites within landscapes with forest cover that was less than 50% contained a different bird species composition than more extensively forested sites and had fewer forest-specialist species and higher beta-diversity. Our study demonstrated the pervasive effect of forest reduction on bird communities in one of the most important hotspots for bird conservation and shows that many vulnerable species require extensive forest cover to persist.

## Introduction

Habitat loss and fragmentation are the major drivers of current rates of biodiversity decline [1]. Although habitat loss generally increases the likelihood of stochastic extinction and declines in population sizes at local and landscape scales, fragmentation effects, i.e., the transformation of the original habitat into a number of isolated fragments in a matrix of habitats that is unlike the original [2], can have positive and/or negative effects depending on species characteristics [1], [3]. Further, although habitat loss and fragmentation are different processes and have different adverse effects on biodiversity, population persistence in anthropogenic landscapes is a result of the interaction of both processes [4], [5].

Ecological studies have shown that the relationship between habitat loss at the landscape scale and extinction of species can be nonlinear [6–8]. The extinction threshold hypothesis states that many species require a given amount of suitable habitat to persist in the landscape. Fragmentation has its most pronounced effects at values that are below this threshold and can lead to abrupt decreases in species population size [4], [9], [10]. Extinction thresholds are proposed to occur when less than 30% of habitat remains, due to a decrease in mean patch size and to an exponential increase in the distance between patches [4], [8]. Attempts to uncover the relative importance of fragmentation and habitat amount have proved a difficult task particularly because there is generally high correlation of most fragmentation metrics to habitat loss, but empirical studies have identified habitat amount as the prevailing driver of species loss [8], [11].

The concept of extinction thresholds was primarily derived from simulations of population responses to habitat loss in neutral landscapes, and current empirical studies have focused more on populations than on communities [4], [12]. The existence of thresholds in communities in response to habitat loss has not always been supported by the published results of empirical studies and is still controversial [12–14]. Threshold values for remaining habitat that range from 5% to 90% have been documented [12], [15], [16]. Such variation might be due to species characteristics, the different measures used to test thresholds (e.g., habitat amount, patch isolation and patch size), the duration and intensity of changes in the landscape, the nature of the matrix and the spatial scale of the studies [9], [14], [17]. Thresholds can also vary among study regions for the same species [18]. Establishing threshold values for an entire community is especially difficult because of the idiosyncratic responses of ecologically different species to habitat loss and landscape structure [19]. Environmental disturbance and changes in habitat quality may decrease the population size of habitat-specialist species but favor an increase of generalist species [20]. Species richness values could therefore be maintained despite variation along the disturbance gradient, such as variation in habitat loss [21].

Responses may vary according to specific ecological traits (e.g., body mass, home range size, migratory status and habitat affinity) [22], [23], even among those groups of species, such as forest-specialist birds, that are usually considered to be sensitive to anthropogenic disturbance. Some studies have highlighted the importance of dietary niche and trophic level as factors that influence the sensitivity of species to disturbed landscapes [23], [24]. Specific trophic guilds, such as understory insectivorous birds [25] and large frugivores [26], are likely to be the first groups to decline in forest landscapes with a reduced amount of habitat. However, the proneness to extinction of even sensitive species varies. For example, frugivorous species show a greater capacity for dispersal and a greater ability to use complementary habitats to obtain food [27] compared with insectivorous species, which require specific local forest characteristics [28–30]. These declines in specific ecological groups can lead to further changes in ecosystem functions in the remaining natural patches [31], [32]. For example, a decline in insectivorous birds may trigger overall changes in trophic cascades [33], the extinction of some frugivorous species may change patterns of seed dispersal [27], and the disappearance of nectarivorous species can lead to a decrease in gene flow among plants, which can then become more susceptible to stochastic extinctions [34].

To address the challenges of preventing biodiversity loss and maintaining ecosystem functioning in human-altered landscapes, it is important to understand how birds that play different ecological roles are affected by habitat loss [35], [36]. In a context of nonlinear relationships, understanding how and where thresholds can occur provides insights to guide landscape planning, management and conservation [37]. In this study, we used an *a priori* classification of bird species according to the available published data and expert opinion, that is based on their dependence on forest (forest-specialists and habitat generalists) and on the specificity of their food resources (frugivorous and insectivorous) to evaluate the responses of these groups to forest cover reduction at 40 forest sites in landscapes that have remaining forest cover that ranges from 6% to 85%. The study was conducted in anthropogenic landscapes in the Brazilian Atlantic Forest, a biome that is highly deforested and disturbed but that still possesses high levels of species richness and endemism [38]. We tested four hypotheses. (i) For all of the species combined, the overall species richness and abundance of birds would not be affected by reductions in forest cover at the landscape scale because of the highly idiosyncratic responses of species of different ecological groups. (ii) The richness and abundance of species of the different groups would vary, e.g., forest-specialist birds would show a strong negative response to forest reduction, generalists would respond positively, and both groups would show nonlinear responses with specific threshold values. (iii) There would be a more abrupt decrease in the species richness and abundance of insectivorous birds that would be triggered at lower levels of habitat loss than there would be for frugivorous species. Previous studies emphasize that habitat loss can be extremely damaging to insectivorous forest birds, due to their low dispersal ability, and habitat and diet specificity [25], [26], [28]. Therefore, if both guilds are nonlinearly affected by habitat loss at the landscape scale, threshold values for insectivorous species are most likely to be higher than those for frugivorous species. We finally expect that (iv) bird communities of different ecological groups would have different species compositions in landscapes with low forest cover. Changes in species composition may occur at high levels of habitat loss due to drastic reduction in species richness (extinction threshold), which will form a subset of species able to survive in disturbed landscapes [39].

## Materials and Methods

### Study area

**Fig 1:**
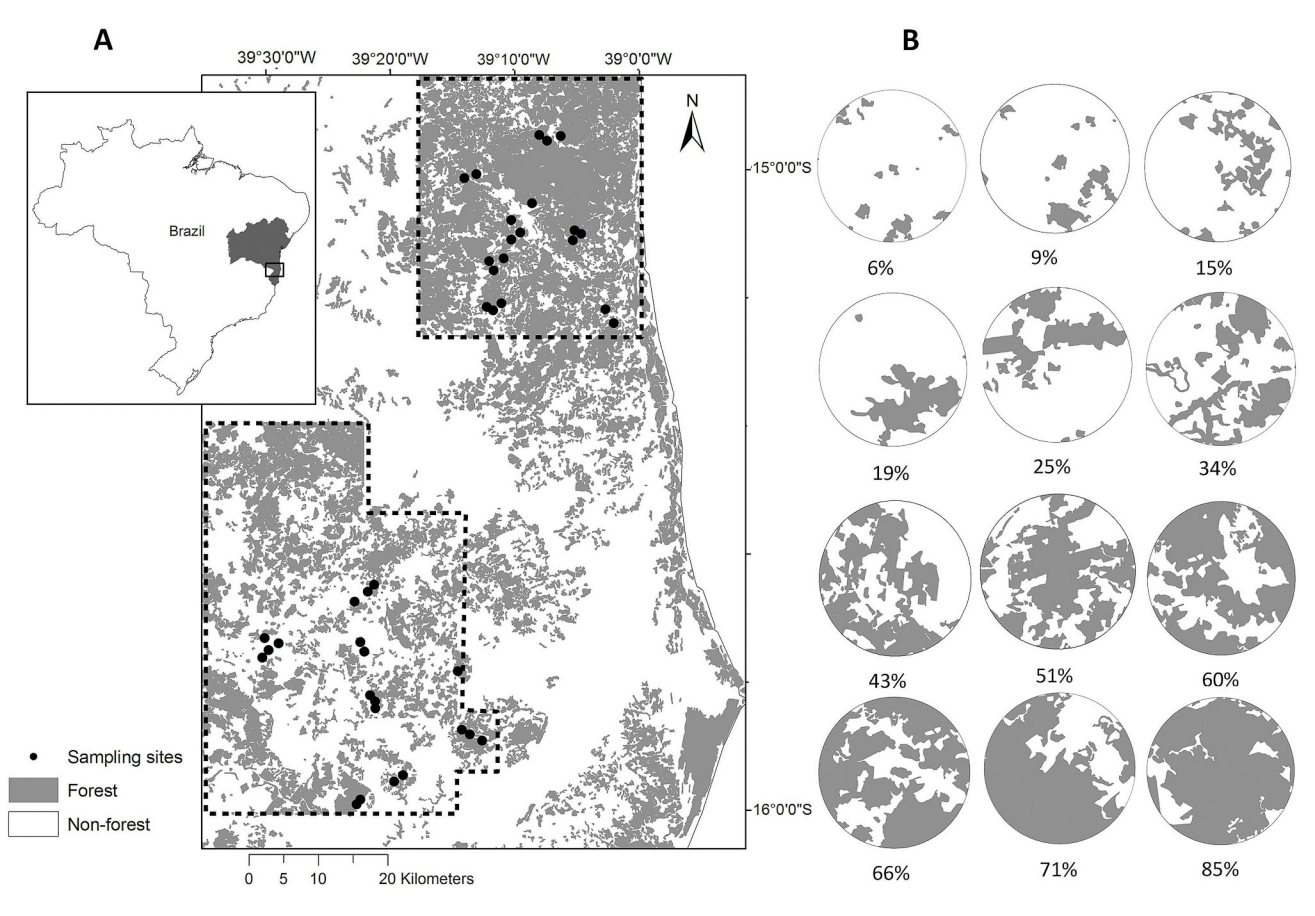
Map of the study area in southern Bahia, northeastern Brazil. A: Atlantic Forest remnants (gray areas) and the 40 sampling sites (black circles). Dashed lines show the areas that were mapped for this study. Images of areas that are outside of the dashed lines were obtained from forest cover map “Atlas dos Remanescentes Florestais da Mata Atlântica” of open access [87]. B: Detail of some sampled landscapes (2 km radius), highlighting the percentage of forest cover (gray areas).

This study was conducted in southern Bahia State, northeastern Brazil (Fig 1). This region is a mosaic of forested habitats that includes remnants of mature forests, secondary forests at different successional stages, shade plantations of cacao (*Theobroma cacao*), rubber trees (*Hevea brasiliensis*) and *Eucalyptus* spp. [20]. The dominant vegetation is classified as Lowland Wet Forest and is characterized by a clear vertical stratification into lower, canopy (25–30 m) and emergent layers (up to 40 m); an abundance of epiphytes, ferns, bromeliads and lianas; and high levels of endemism of different groups [40], [41]. The average annual temperature is 24°C, and the mean annual rainfall is 1500 mm. There is no defined seasonality, although a rainless period may occur from December to March [42].

### Sampling design

This study is part of REDE SISBIOTA, a major research network designed to investigate how the reduction of forest cover affects regional biodiversity patterns and processes in anthropogenic landscapes. We had previously identified a region between the Jequitinhonha and Contas Rivers that still harbor large, representative forest tracts, and these forests have similar soil, topography and floristic composition [40].

We mapped this region by analyzing satellite images that were specifically acquired for our work (QuickBird and WorldView, from 2011) or were already available (RapidEye, from 2009–2010). The mapping was created by manually digitizing the land cover features visually interpreted at scale of 1:10000, which is adequate for identifying patches based on the visual inspection of differences in color, texture, shape, location and context. Patches were delimited as polygons, and a digital map was created using ArcGIS software. Polygons were classified according to different forest types following the typologies provided by IBGE [43]. After intensive ground-truthing, we developed a map of the land use of a 3500 km^2^ area that encompasses the municipalities of Belmonte, Una, Santa Luzia and Mascote. The coordinates of the center of the sampled area are 15° 28’S and 39° 15’W. At a regional scale, there was a north-to-south gradient in forest cover within the mapped region (Fig 1). Although there are open areas within the mapped region, most of the large and continuous forests in the northern area are concentrated around the Una Biological Reserve and the Una Wildlife Refugee, two federally protected conservation units that have a total area of 34804 ha, which includes the municipality of Una. In contrast, the southern part of the mapped area is clearly more deforested than the northern part, but there are still some large forest tracts in the southern part.

Based on this map, we identified 58 potential sampling sites that were located in forest patches. We adopted the patch-landscape approach [44], in which the response variables are evaluated within forest patches, and the landscape variables are measured within a specific area surrounding the each sampling site. To characterize the landscape, we quantified the percentage of forest cover using ArcGIS software within a 2 km radius from the center of each sampling site (which yields a surface area of each site of approximately 13 km^2^). We considered only native forests in our estimations of the amount of forest cover within the landscape. Therefore, forest cover included all of the native forest types, encompassing the mature and successional forests types described above but excluding shade plantations of cacao and rubber trees. This classification may be a simplification of the ecological requirements of bird species, but we believe that this broad definition is the most appropriate because many recommendations for the conservation and management of landscapes are based on fragmentation or habitat loss in general [45].

We excluded those sites that were located at a distance of less than 1 km from the closest site to avoid recounting individuals that have high dispersal ability and large home ranges (e.g., falcons and parrots). We randomly selected 40 sites that had 6% to 85% forest cover within a radius of 2 km. Twelve sites had 6% to 30% forest cover, 13 sites had 31% to 50%, and 15 sites had 51% to 85%. The distance between sites ranged from 1 to 105 km. We did not sample in either of the protected areas, and no specific permission was required for the selected locations. However, we secured permission to conduct fieldwork in all sampling sites that were located on private land.

### Bird survey

We sampled bird communities in three field campaigns: January to April 2013, May to September 2013, and October 2013 to April 2014. The climatic conditions during the campaigns did not affect the sampling of birds, since there is no seasonality defined in the study region [42]. Moreover, each site was sampled once during the bird breeding season (September to January) to avoid any bias, since the birds are more active this period of the year.

We used the point-count method [46], and at each sampling site we established four sampling points that each had a radius of 50 m and that were separated by a distance ranged from 150 to 550 m [46]. We assigned sampling points inside each forest area that were at least 100 m from the edge to avoid effects of adjacent habitats and to ensure that the documented bird community was representative of the site.

All sites were covered in each field campaign, and sampling at each point was conducted for 15 min at sunrise (between 0600 and 0900 hr) and at sunset (between 1500 and 1700 hr), which are the periods of greatest bird activity. Therefore the sampling effort at each sampling site was 6 hours. We recorded each bird that was seen or heard at each sampling point. We avoided sampling on rainy and windy days because such conditions reduce bird detectability [46]. We excluded birds that were flying over the forest and birds that could not be located precisely.

We used 8x42 binoculars to identify the birds and a digital recorder to record their vocalizations. We confirmed vocalization-based bird identifications by playback or by comparing the recordings with an existing database. Field guides [47], [48] were used for identification. The scientific nomenclature used conforms to that of the South American Classification Committee [49].

### Data analysis

We designated bird communities as forest-specialist and generalist species based on the scientific literature [23], [50]. The endemic birds of the Atlantic Forest and those that occur in forested habitats of the Atlantic and Amazon Forests, according to Stotz et al. [50], were classified as forest-specialist species. Species that also occur in open vegetation habitats, such as grasslands, of the Cerrado, Caatinga, Pampa and anthropogenic areas were classified as generalists. The forest-specialist species were also grouped according to their trophic guild (i.e., insectivores, frugivores, nectivores, omnivores, carnivores, and granivores). Trophic categories reflect the main food source of the species, and birds were categorized as omnivores if their diet is composed of different classes of food items. These classifications were based on our prior knowledge about the ecology of the species, information available on the literature and after consulting specialists.

We first evaluated the effect of variation in bird diversity based on biogeographical factors by means of a Mantel test between the geographical distance matrix and two matrices of differences in species richness and abundance between pairs of sampling sites. We then assessed the relationship between the number of species (richness) and the total number of individuals (abundance) of the most representative groups (overall species, generalists, forest-specialist, forest frugivores and forest insectivores) and forest amount in the 40 sites. Total richness and abundance in each site were considered as the sum of the number of species and individuals, respectively, recorded during the three field campaigns in the four counting points. We used a null model, a generalized linear model and a logistic model with four parameters to evaluate the bird response types (linear and nonlinear). We assumed a Poisson error distribution for the abundance and species richness data in each of the models.

Null models were used to test the absence of effects, and GLMs were used to test the existence of a continuous change in the biological response related to forest cover. The four-parameter logistic regression, which is expressed in the formula $F\left(x\right)\;=\;d+(\frac{a}{1+e\left(\left(b-x\right)\right)})$, is a nonlinear model that has a sigmoidal shape that is appropriate to fit to threshold curves [51]. This model has a lower asymptote (d), which is the lower value of the response variable, and an upper asymptote (a + d). The parameter “a” represents the difference in the response variable before and after the decay phenomena expressed in the model, and “b” is the inflection point, the point at which the curve tends to change from one asymptote to another. The parameter "c" is proportional to the slope of the ascending part of the curve or to the speed at which it reaches the asymptote near the inflection point "b" [51], [52].

The models were subjected to model selection using models’ Akaike weights, calculated using Akaike information criterion corrected for small sample sizes (AICc) [53]. The AICc weights or model probabilities (ranging from 0 to 1), express the normalized relative likelihood of each model. Models that present Akaike weights with more than half the value of the best model (higher weight) was considered to further investigation. After model selection, we analyzed the residual distributions of the best models and the confidence intervals of the parameters.

In the case where the most likely relationship was represented by nonlinear models, we used piecewise models to determine the inflection point correspondent to extinction threshold values. A piecewise model identifies two or more straight lines that are joined at an unknown point, called the breakpoint [52], and can be considered the indicator of the bird extinction threshold [54], [55].

We used nonmetric multidimensional scaling (NMDS, two axes) to analyze differences in bird communities among landscapes. We used presence-absence data and the Jaccard similarity index to perform an ordination of landscapes that was based on their similarities in species composition. An analysis of similarities (ANOSIM) was performed to test for differences in bird composition between landscapes that had amounts of forest cover that were below and above the threshold values that had been determined with the piecewise model. The NMDS and ANOSIM analyses were performed for the most representative bird groups (generalists, forest-specialist, forest frugivores and forest insectivores). We also performed direct gradient analysis [56] that used presence-absence data to verify the replacement in bird species along the gradient of forest cover and to determine which species occur in landscapes that are located below and above the extinction threshold. All the statistical analyses and graphs were carried out in R software [57] using vegan [58], mass [59], nlme [60], bbmle [61] and segmented [62] packages, with an adopted alpha of ≤ 0.05 considered significant. Custom R scripts for the analyzed data are provided in S1 File.

## Results

### Bird community

The total sampling effort involved 240 hours that were equally distributed among sampling sites. We recorded 5931 individuals that belonged to 184 species and 39 families at the 40 sampling sites. The families Tyrannidae (19 species, n = 889), Thraupidae (17 species, n = 697) and Thamnophilidae (15 species, n = 724) had the greatest abundance and species richness. The species with the greatest abundance were *Cacicus cela* (n = 238), *Tolmomyias flaviventris* (n = 173), *Machaeropterus regulus* (n = 163) and *Thamnophilus ambiguus* (n = 159). Only one individual was recorded for each of 11 other species (*Celeus torquatus*, *Coccyzus euleri*, *Cyanerpes cyaneus*, *Euphonia cyanocephala*, *Myrmotherula minor*, *Myiothlypis rivularis*, *Anabacerthia lichtensteini*, *Pionus menstruus*, *P*. *maximiliani* and *Sclerurus mexicanus*). These birds were observed mainly in landscapes that had high forest cover. In contrast, species such as *Patagioenas speciosa*, *Phaethornis ruber* and *T*. *flaviventris* were frequently observed in landscapes that had different amounts of forest cover. These species were recorded in 37, 35 and 33 landscapes, respectively.

Overall, approximately 60% of birds were forest-specialist species (103 species, n = 3715). Insectivorous birds showed the greatest richness (56 species, n = 1935), followed by frugivores (34 species, n = 1165). The other trophic guilds were poorly represented (Table 1) and therefore were not used in the analyses.

**Table 1:** Richness and abundance of birds of different ecological groups.

Ecological groups	Richness	Abundance
Generalist	81	2216
Forest-specialist	103	3715
**Trophic guilds of forest-specialist**		
Frugivorous	34	1165
Insectivorous	56	1935
Omnivorous	6	452
Nectarivorous	3	136
Carnivorous	3	13
Granivorous	1	14

### The effect of forest cover reduction on the bird community

**Fig 2:**
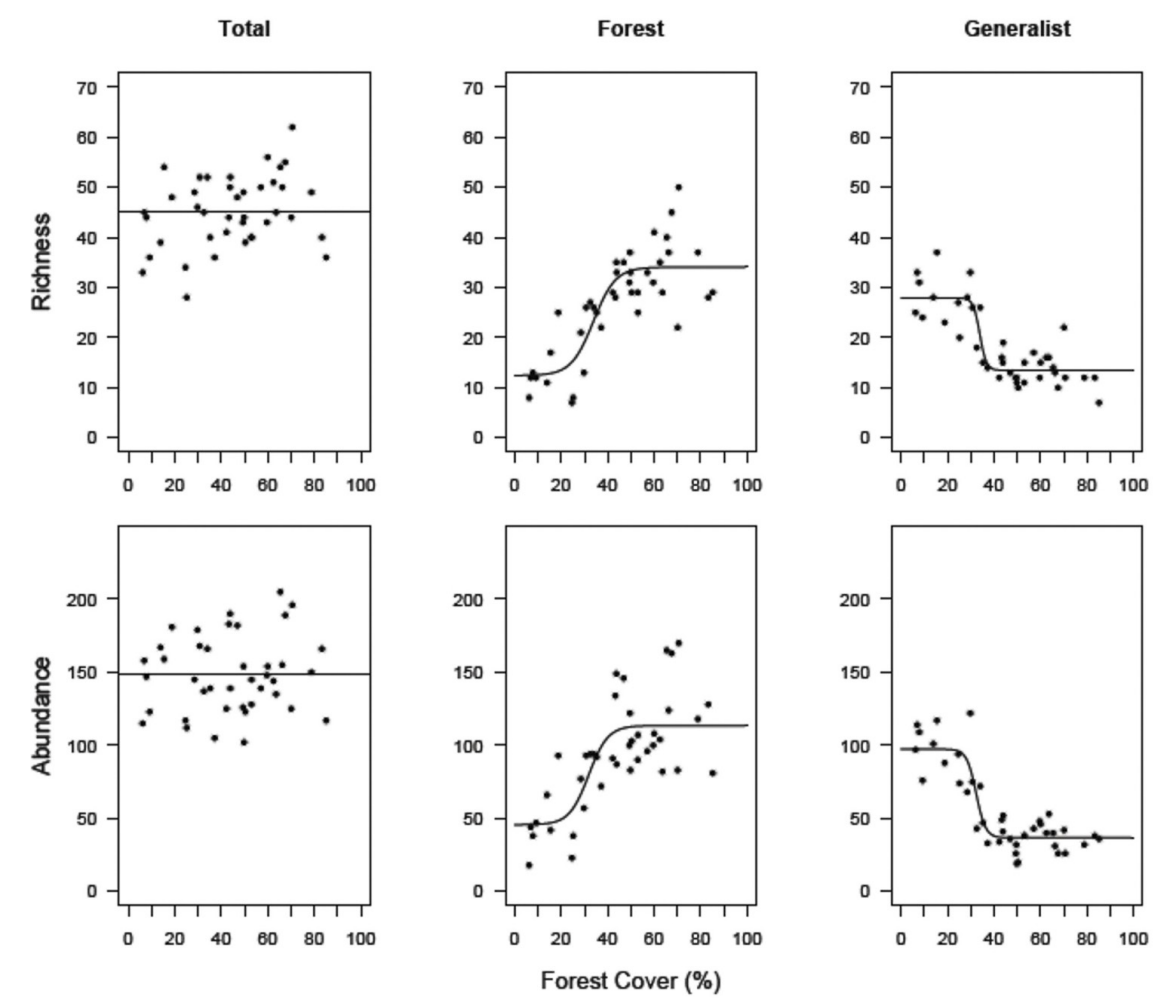
Total richness and abundance of forest-specialist and generalist species in the 40 sampling sites. Landscapes vary in the amount of remaining forest cover from 6% to 85%. Lines correspond to the best fitting models.

We found no spatial correlation between geographical distances and differences in species richness (r = 0.05, p = 0.07) and abundance (r = 0.009, p = 0.30) among sampling sites. The greatest richness (62 species) and abundance (205 individuals) were observed in landscapes with 71% and 65% of forest cover, respectively, whereas the poorer (28 species) and less abundant sites (102 individuals) were observed in sites with 25% and 50% landscape scale forest cover, respectively. Overall abundance and species richness were not affected by differences in forest cover at the landscape level (Fig 2). Although the AICc weight showed that GLMs were the best models (Table 2), its correlation coefficients were very low (0.002 for richness and 0.0009 for abundance). This finding indicates that the models showed straight lines that were almost parallel to the x axis, which is very similar to the null models. Therefore, both models showed the lack of relationship between the dependent variables and forest cover.

**Table 2:** Best models (in bold) for explaining the relationship between richness and abundance of ecological groups of birds and the amount of forest cover.

	Richness					Abundance				
Species group	Model	AICc	Δ_i_	k	w_i_	Model	AICc	Δ_i_	k	w_i_
Total	**GLM**	**270.91**	**0**	**2**	**0.64**	**GLM**	**457.59**	**0**	**2**	**0.49**
	**NULL**	**272.58**	**1.67**	**1**	**0.27**	**NULL**	**457.79**	**0.2**	**1**	**0.46**
	FLM	275.03	4.12	4	0.09	FLM	462.56	4.97	4	0.04
Forest-specialist	**FLM**	**264.57**	**0**	**4**	**1**	**FLM**	**526.84**	**0**	**4**	**1**
	GLM	287.97	23.4	2	<0.01	GLM	606.03	79.2	2	<0.01
	NULL	380.62	116.1	1	<0.01	NULL	889.98	363.1	1	<0.01
Generalists	**FLM**	**226.27**	**0**	**4**	**0.98**	**FLM**	**350.09**	**0**	**4**	**1**
	GLM	233.69	7.5	2	0.02	GLM	420.67	70.6	2	<0.01
	NULL	305.53	79.4	1	<0.01	NULL	789.34	439.2	1	<0.01
Frugivores	**FLM**	**174**	**0**	**4**	**0.99**	**FLM**	**405.92**	**0**	**4**	**1**
	GLM	184.69	10.7	2	0.01	GLM	447.87	42	2	<0.01
	NULL	216	42	1	<0.01	NULL	598.69	192.8	1	<0.01
Insectivores	**FLM**	**236.56**	**0**	**4**	**0.99**	**FLM**	**475.9**	**0**	**4**	**1**
	GLM	250.2	13.6	2	0.01	GLM	517.74	41.8	2	<0.01
	NULL	304.65	68.1	1	<0.01	NULL	657.63	181.7	1	<0.01

Models: Null model (NULL), generalized linear model (GLM) and logistic model with four parameters (FLM). AICc: Akaike information criterion corrected; Δ_i_: difference in AICc between the best model and the ith model; k: parameter number of the model; w_i_: AICc weight. Models are ranked by AICc values.

The effect of forest cover was evident when the species were classified into *a priori* ecological groups (Table 2). Forest-specialist bird diversity showed a nonlinear relationship and was negatively affected by a reduction in forest cover at the landscape scale (Fig 2). The piecewise model showed that an abrupt decrease in forest-specialist diversity occurs in landscapes that have an amount forest cover that is less than 46%±3.9% (for richness) and 44%±2.2% (for abundance). Conversely, forest cover reduction positively affected generalist birds, with significant nonlinear responses of species richness and abundance along the gradient of forest cover. There was a rapid change in the richness of generalist birds when the amount of forest at the landscape scale reaches 50%±10.2% (Fig 2). There was a decline in the diversity of bird generalists in landscapes that have an amount of forest cover that is above this value, and landscapes with less forest cover have more generalists. Additionally, specific threshold values were quite similar for generalist abundance (49%±4.3%).

**Fig 3:**
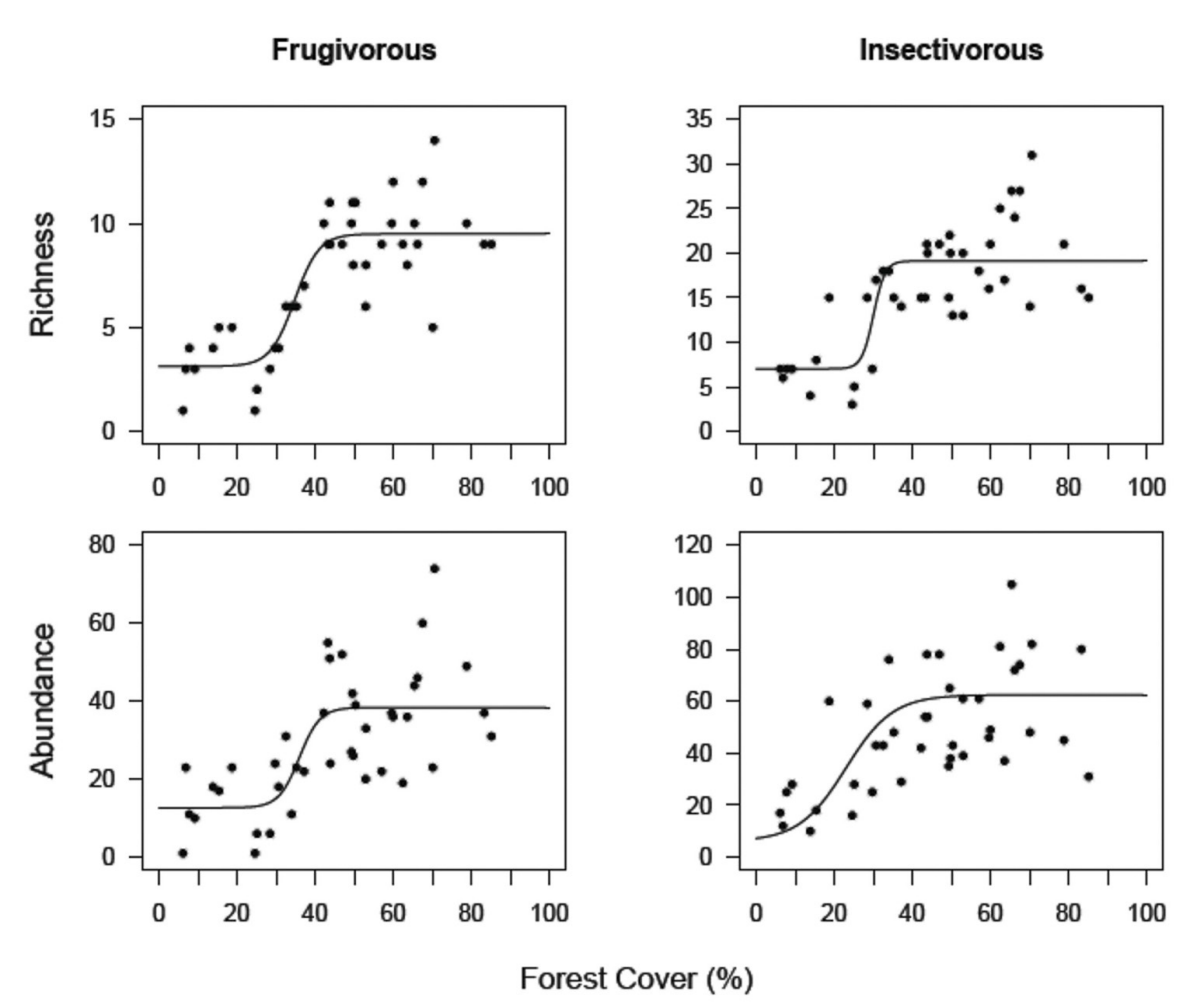
Richness and abundance of frugivorous and insectivorous birds in the 40 sampling sites. Landscapes vary in the amount of remaining forest cover from 6% to 85%. Lines correspond to the best fitting models.

As with forest-specialist species, forest frugivores and insectivores were also negatively associated with forest cover. The abundance and species richness of both groups declined in a nonlinear pattern (Fig 3). Piecewise models indicated thresholds of loss of frugivorous species and individuals respectively at 46%±5.4% and 44%±3.7% of forest cover at the landscape scale. For insectivorous birds, the extinction threshold for richness occurred in landscapes with 44%±4.9% forest cover, and abundance decreased quickly in landscapes with forest cover of less than 34%±2.6%. There was substantial variation in the number of individuals (i.e., abundance) of both groups along the forest cover gradient. Landscapes with similar amounts of forest cover sometimes had different bird abundance. For example, 37 insectivorous birds were counted in a landscape with 63% forest cover and 105 insectivorous birds were counted in a landscape with 65% forest cover (Fig 3).

### Change in bird species composition

**Fig 4:**
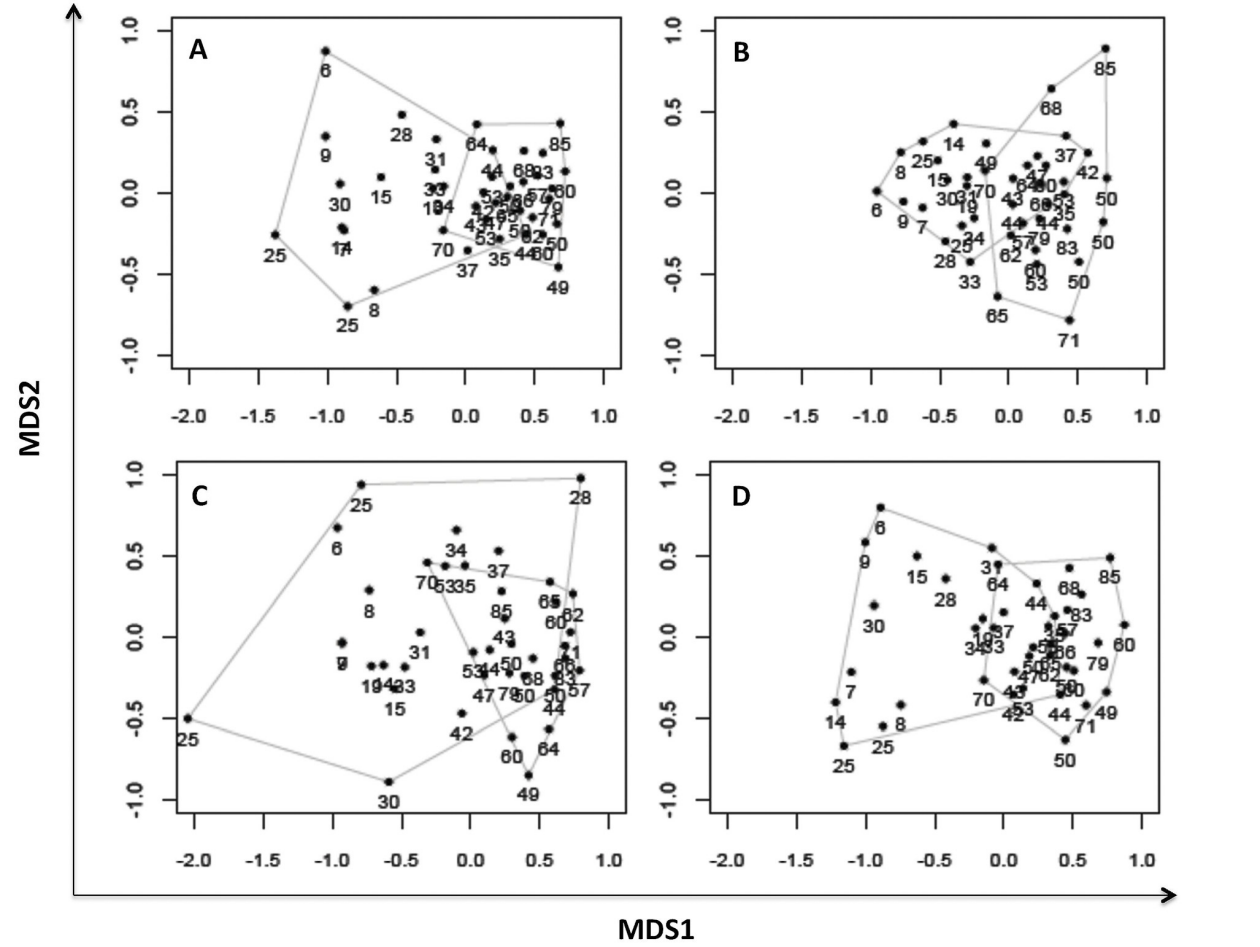
NMDS ordination of the 40 sites that were sampled in the Brazilian Atlantic Forest. Black points represent the scores of landscapes and the numbers indicate the percentages of forest cover (from 6% to 85%). A: Forest-specialist birds, B: Generalist birds, C: Frugivorous birds, D: Insectivorous birds. Pairwise ANOSIM tests showed significant differences (p < 0.05) between the bird compositions of landscapes with percentages of forest cover that were less than (left polygons) and greater than (right polygons) the threshold values.

The bird species composition of all ecological groups was also affected by forest cover. The first two axes of the NMDS had a stress value of 0.15 for forest-specialist, 0.19 for generalists, 0.17 for frugivorous, and 0.16 for insectivorous birds. This finding indicates that our data were represented well in these two dimensions. The two axes of the NMDS showed a clear separation between landscapes (Fig 4). One group was composed of landscapes that had forest cover that was less than the observed threshold values for the different ecological groups (represented by lower scores on the first axis), and another group was composed of landscapes that had forest cover that was greater than the observed threshold values (represented by higher scores on the first axis). Overall, bird species composition among landscapes with low forest cover (below the threshold) showed great dissimilarity (high beta-diversity) compared with those with more forested landscapes and higher variation in scores on the second axis (Fig 4). In addition, comparisons of the ANOSIM analysis showed significant differences in the species composition of forest-specialist (R = 0.39, p = 0.001), generalists (R = 0.28, p = 0.001), insectivores (R = 0.33, p = 0.001) and frugivores (R = 0.36, p = 0.001) in landscapes with an amount of forest cover that was less than, and greater than, the threshold.

The direct ordination showed a replacement of species of different groups with forest cover (S1–S4 Figs). Twenty-six species of forest-specialist occurred exclusively on landscapes with high forest cover (S1 Fig). Eight of these species are frugivorous (e.g., *Carpornis melanocephala*, *Turdus albicollis*, *Xipholena atropurpurea* and *Euphonia pectoralis*), and 16 are insectivorous (e.g. *Drymophila ferruginea*, *Eleoscytalopus psychopompus*, *Formicarius colma* and *Philydor atricapillus*). All of these species disappeared in landscapes with less than 50% of their original forest cover (S3 and S4 Figs). Conversely, from the total of generalist birds, 30 species are favored by the decrease in forest. These species occur exclusively in landscapes with low forest cover (S2 Fig).

## Discussion

### Forest cover and species diversity

We found that forest reduction at the landscape scale triggers major changes in the bird communities that inhabit anthropogenic landscapes in the Brazilian Atlantic Forest. As hypothesized, when all species combined were considered, bird richness and abundance were not affected by forest cover reduction at the landscape scale. This pattern occurred because the bird community was composed of species that have different responses to environmental perturbation. There was species that are not affected by a decrease in forest cover and species that are positively or negatively affected by the change [19]. One can therefore expect that overall richness and abundance are maintained along the gradient of forest cover by the replacement of sensitive bird species by those that are favored by deforestation [63].

The compensatory response of birds was clear when different ecological groups were considered, which indicated that overall richness and abundance can mask striking changes in community patterns and can be misleading as biodiversity indicators of meaningful conservation value [64]. Forest-specialist birds showed an abrupt decrease in species richness in landscapes that had a forest cover that was less than 50%, but there was a concomitant increase in the richness of generalist birds. Our results therefore demonstrated how bird community structure changes when forest is lost. The diversity of forest-specialist birds is maintained when more than 50% of the forest cover remains. However, a decrease in forest cover below this critical value (<50%) creates novel habitats that favor generalist bird species, which may be better adapted to use disturbed habitats [23]. Forest reduction also triggered a major loss in the species richness of frugivorous and insectivorous birds. Frugivorous and insectivorous birds, regardless of their specific ecological characteristics, showed extinction threshold values that were representative of all forest-specialist.

Alpha diversity tended to be lower with progressive habitat loss, and the remaining species assemblages constituted a subset of more tolerant or disturbance-adapted species [65], [66]. By contrast, there was high species replacement (beta diversity) in these deforested landscapes, which helped to maintain relatively rich and abundant bird assemblages in a regional scale (gamma diversity). Further, the species composition of all ecological groups changed in landscapes with reduced amount of forest cover. Thus, habitat loss can act as an environmental filter and select species with ecological traits able to survive in landscapes with reduced amount of forest [39].

The level of functional redundancy among bird species is not obvious, and it is therefore necessary to understand how and whether this clear pattern of species decline and replacement can lead to the loss of ecosystem functioning [67]. For example, the disappearance of frugivorous birds may change seed dispersal patterns and thus affect forest structure [68], and the decline of insectivorous birds may increase the population of herbivorous insects and consequently affect leaf damage and photosynthesis [69].

### Bird extinction threshold

In simulated landscapes that have a low proportion (usually less than 30%) of original habitat, the mean patch size is reduced and, as habitat loss continues, there is an exponential increase in the mean distance between patches [4]. Species extinctions within small patches are not offset by migration among patches in such highly deforested and fragmented landscapes, which triggers a threshold of species extinction [4]. This extinction threshold in landscapes that have less than 30% of remaining habitat has been reported empirically in studies of different taxonomic groups in anthropogenic landscapes in various regions [8], [19], [37], [70]. Within the Atlantic Forest, extinction thresholds that range from 10% to 40% of forest cover have been reported in studies that focused on plants [70], [71] and mammals [8], [72].

However, our results indicated that landscapes that still have a large proportion of forest (~50%) may exhibit a sharp decline in species diversity. Similar results for birds were observed in the southeastern Atlantic Forest [73], which indicates that these effects are not unique to our study. Martensen et al. [73] reported an abrupt decrease in the species richness of sensitive birds when there is less than 50% of forest cover in a landscape. One possible reason for this high threshold value is that most tropical bird communities are composed of rare and specialized species that are more sensitive to alterations in their habitat and therefore require more forest [73]. Indeed, southern Bahia is rich in bird species, even compared with the northern and southern portions of the Atlantic forest, and most of the birds that were observed in the present study were forest-specialist that are often sensitive to forest loss [50].

In a recent conceptual model, Villard and Metzger [65] proposed that extinction thresholds can be influenced by the configuration of the elements that comprise the landscape, with the most vulnerable species being those that have a narrow range within which habitat loss can be mitigated in part by favorable habitat configurations. Although our estimates of forest cover included only native vegetation, the matrix of some of our landscapes also included shade cacao plantations, which is an anthropogenic forest category in which many bird species are reported to occur [20]. It is therefore surprising that, even in landscapes that have such relatively permeable matrices as shade plantations, a large amount of native habitat is still required to maintain different ecological groups of birds. It is also important to highlight that the habitat categorization that we used may have influenced the threshold values. We used the total of all forest types in different successional stages when calculating the percentage of remaining forest. Previous studies conducted in the region [20], [74], [75] document that the different categories of native forest mosaics contain different species communities. It is possible that the amount of forest that is effectively used by forest-specialist species is less than the amount of forest that is actually available in the landscape. However, it may be impossible in empirical analyses to quantify the conditions that limit the occurrence of every species [76]. This is particularly true in neotropical regions because of their high species diversity and inadequate scientific knowledge of the ecological requirements of the birds.

All ecological groups of birds showed nonlinear responses to the relationship between abundance and forest cover reduction. The abrupt decreases in abundance that follow small changes in the amount of forest cover can be extremely important for conservation. Even when certain species are present in landscapes that have an amount of forest that is less than the observed threshold, their density may be so low that the species is functionally extinct, which is a stage that precedes the actual extinction of the species [77], [78]. Additionally, frugivorous and insectivorous birds showed large variation in abundance in landscapes that had similar amounts of forest. This variability may indicate that there was random variation or that there are other factors that are important for maintaining populations of these species. Insectivorous birds, especially those that use the understory, have a low capacity for dispersal and are affected by local modifications of vegetation structure [25]. Local characteristics of a forest can therefore be as important as variables at the landscape scale. Frugivores depend on seasonal resources and must therefore move daily to obtain food and are likely to rely on the use of multiple habitats [79]. However, the degradation of natural habitats may lead not only to habitat loss but also to a simplification of the matrix structure, which makes the landscape less permeable to species movement [80]. An inhospitable matrix and increasing distance between patches can impede species dispersal because of higher energetic demand and high predation risk [27], which would lead to a low abundance of frugivores in some landscapes [35], [81]. Although shade plantations provide complementary habitats for a variety of bird species in our region [82], these agroforests may negatively affect insectivorous and frugivorous birds that live in the understory because the native understory is completely replaced by cacao plants.

### Implications for conservation

The use of extinction thresholds can be an important tool to help natural resource manager to biodiversity conservation [17]. Identifying thresholds, it is possible to propose appropriate management of the landscape to maintain or restore forest cover values above that threshold, which is more likely to retain a greater species diversity [83], [14].

Current Brazilian environmental laws require that the amount of protected areas within the Atlantic forest domain be equivalent to 20% of the total area of private rural properties [84]. However, even assuming that property-scale habitat amount could somehow reflect overall landscape-scale spatial patterns, extinction threshold values that were found in the present study indicate that more forest should be protected to ensure the persistence of most habitat-sensitive birds, such as forest-specialists, frugivores and insectivores. Bird species belonging to those groups require that approximately 50% of a given site be occupied by protected forest to maintain their diversity. The agroforestry systems that are present in the study region do provide complementary habitats for many species [20], [74], [82], and can therefore mitigate the effects of habitat loss at some extent, but many of the bird species sampled here are very habitat specific, thus exclusively depending on native forest habitats to survive. Currently, the remaining forest cover of the Brazilian Atlantic Forest is only 11% of its original extent [85]. The best preserved areas are located in the southern states at Serra do Mar, which has 36.5% of its original vegetation, and the remnants that still exist in Bahia State (17.7%) [85]. These values suggest that there is an urgent need for forest restoration policy at both state and national scales to ensure that there is enough forest to conserve bird diversity dependent on forested environments [86].

## Supporting Information

S1 Fig Occurrence of forest-specialist birds in relation to the amount of forest cover in the 40 sampling sites. The vertical line indicates the threshold value estimated by the piecewise model.
(TIF)

S2 Fig Occurrence of generalist birds in relation to the amount of forest cover in the 40 sampling sites. The vertical line indicates the threshold value estimated by the piecewise model.
(TIF)

S3 Fig Occurrence of frugivorous birds in relation to the amount of forest cover in the 40 sampling sites. The vertical line indicates the threshold value estimated by the piecewise model.
(TIF)

S4 Fig Occurrence of insectivorous birds in relation to the amount of forest cover in the 40 sampling sites. The vertical line indicates the threshold value estimated by the piecewise model.
(TIF)

S1 File Commands executed in R Program to conduct the data analysis of bird ecological groups. (DOCX)
