# Supplementary figures and images for: Birds in Anthropogenic Landscapes: The Responses of Ecological Groups to Forest Loss in the Brazilian Atlantic Forest
Source: PLoS One. 2015 Jun 17;10(6):e0128923. doi: 10.1371/journal.pone.0128923 (PMC4471271; doi:10.1371/journal.pone.0128923)

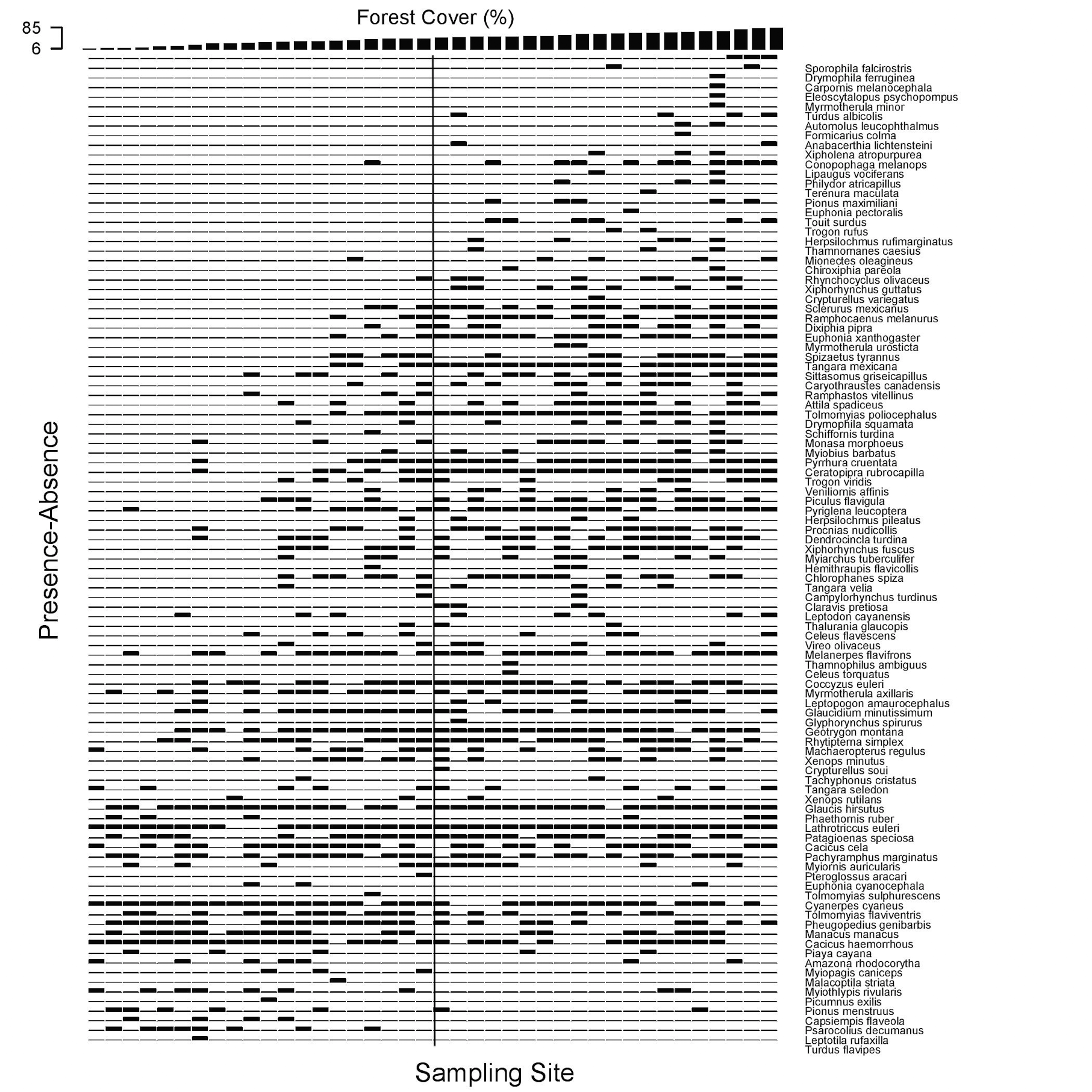

Supplement: S1 Fig — The vertical line indicates the threshold value estimated by the piecewise model. (TIF) [file pone.0128923.s001.tif]

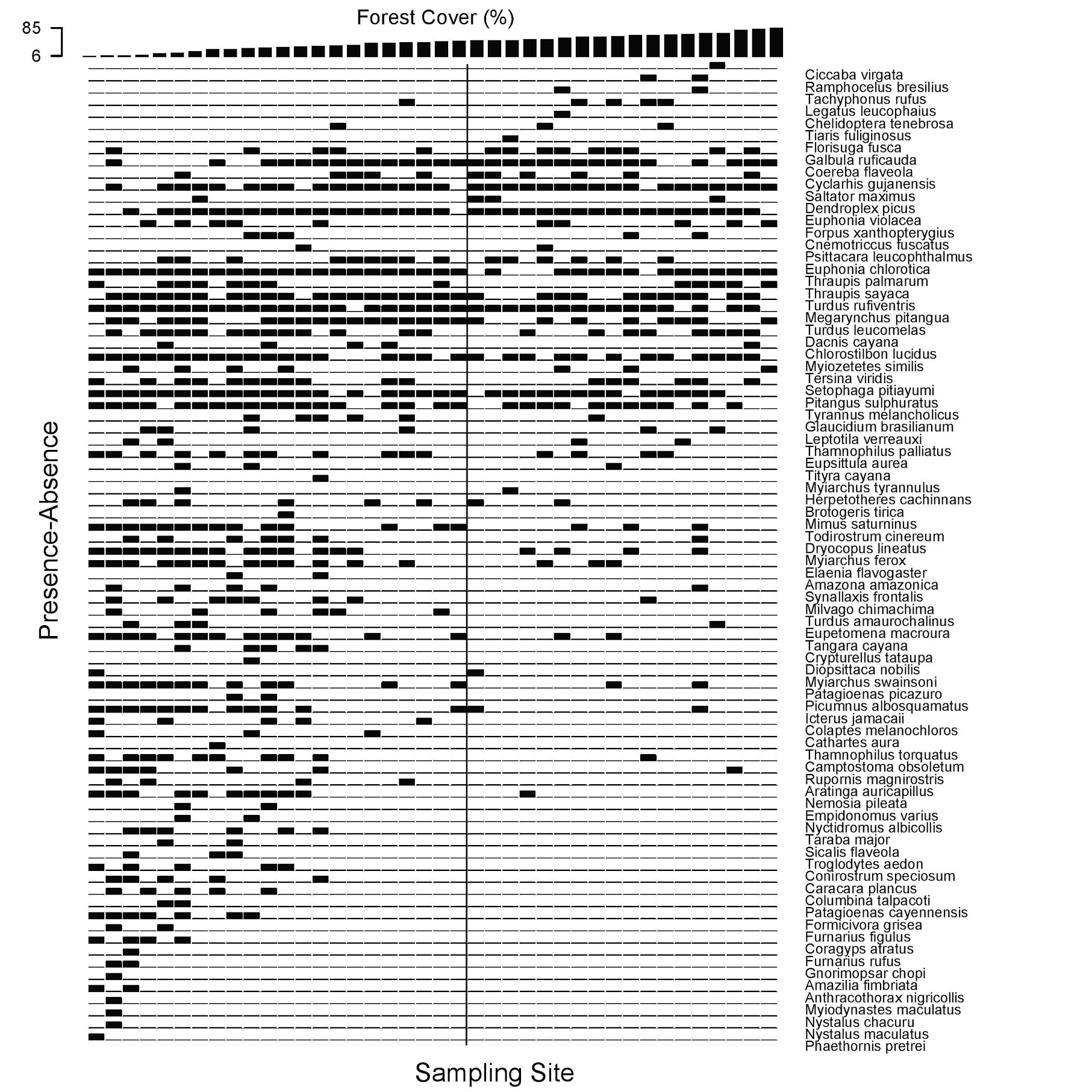

Supplement: S2 Fig — The vertical line indicates the threshold value estimated by the piecewise model. (TIF) [file pone.0128923.s002.tif]

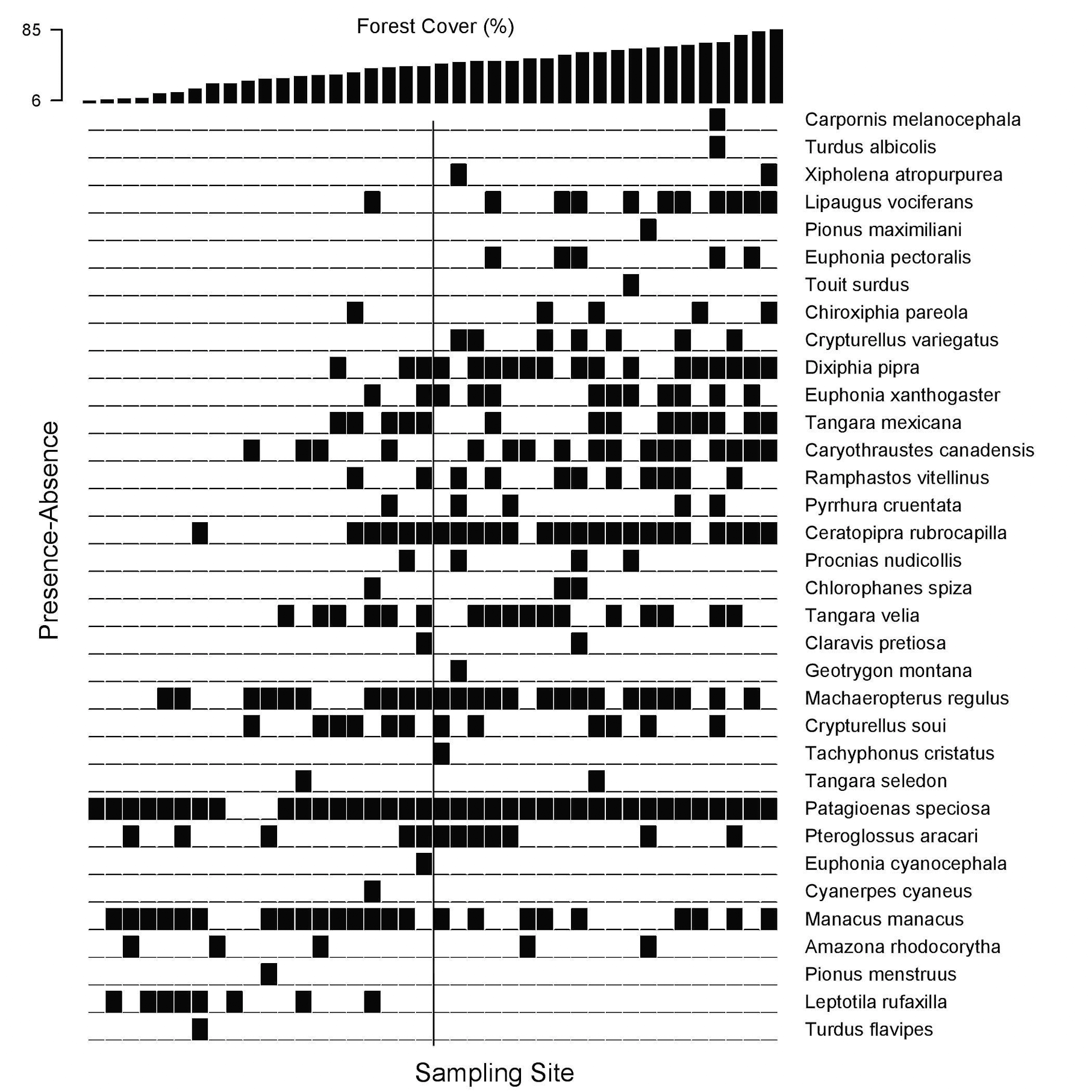

Supplement: S3 Fig — The vertical line indicates the threshold value estimated by the piecewise model. (TIF) [file pone.0128923.s003.tif]

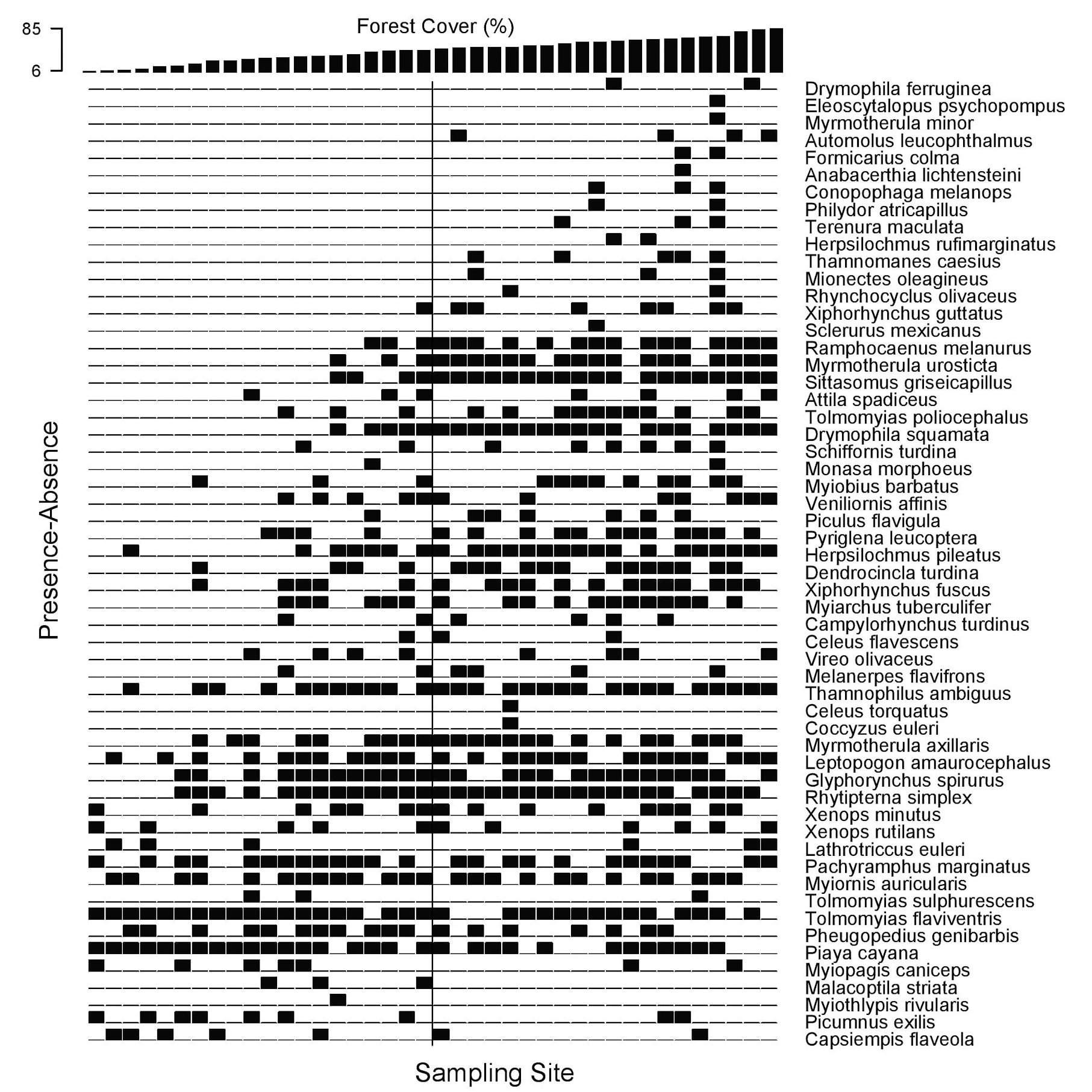

Supplement: S4 Fig — The vertical line indicates the threshold value estimated by the piecewise model. (TIF) [file pone.0128923.s004.tif]
