# Supplementary material for: Birds in Anthropogenic Landscapes: The Responses of Ecological Groups to Forest Loss in the Brazilian Atlantic Forest
Source: PLoS One. 2015 Jun 17;10(6):e0128923. doi: 10.1371/journal.pone.0128923 (PMC4471271; doi:10.1371/journal.pone.0128923)
Supplement: S1 File — (DOCX) [file pone.0128923.s005.docx]

**Supplementary Data: R Script for conducting bird richness and abundance analysis**

#packages

library("vegan")#[1]

library("MASS")#[2]

library(nlme)#[3]

library("bbmle")#[4]

library("segmented")#[5]

#loading table

Tabela_aves=read.table("c:/data frame/Tabela_aves.txt", header= T, sep="")

#loading variables

#total richness of birds

tot.rich=Tabela_aves$Riq_total

#total abundance of birds

tot.abu=Tabela_aves$Abund_total

#generalist birds richness

gen.rich=Tabela_aves$Riq_generalista

#generalist birds abundance

gen.abu=Tabela_aves$Abund_generalista

#forest-dependent birds richness

for.rich=Tabela_aves$Riq_florestais

#forest-dependent birds abundance

for.abu=Tabela_aves$Abund_florestais

#frugivorous birds richness

fru.rich=Tabela_aves$Riq_FRU

#frugivorous birds abundance

fru.abu=Tabela_aves$Abund_FRU

#insectivores birds richness

ins.rich=Tabela_aves$Riq_INS

#insectivores birds abundance

ins.abu=Tabela_aves$Abund_INS

#forest cover

cover=Tabela_aves$Por

############

#**First - Evaluated the bird response types (linear and nonlinear)**

**#Models: total richness**

#Generalized Linear model

modTR.glm=glm(tot.rich~cover, family=poisson)

#four parameter logistic regression with poisson error

logip=function(p,lambda,x){

a=p[1]

b=p[2]

c=p[3]

d=p[4]

Riq1 = d+(a/(1+exp((b-cover)/c)))

-sum(dpois(x,lambda=Riq1, log=TRUE))

}

parnames(logip)=c("a","b","c","d")

modTR.log=mle2(minuslog=logip, start= c(a=30,b=30, c=3,d=20), data=list(x=tot.rich))

#null model

modTR.null=glm(tot.rich~1, family=poisson)

#model selection

AICctab(modTR.glm,modTR.log, modTR.null, weights=T, nobs=40)

##null and glm are almost equiprobable, let us see the points, the models and residuals

#plots:

plot(cover,tot.rich, xlab="Forest cover", ylab="Total Richness")#original data

abline(h=mean(tot.rich))#null model

points(cover, modTR.glm$fit, type="l", col="dark gray")##glm model

#investigating the glm parameters

coef(modTR.glm)

#a very low value of correlation coeficient.

#null model explins better

#residual analysis

plot(modTR.glm$res, cover)

plot(modTR.null$res, cover)

##########

**#Models: total abundance**

#Generalized Linear model

modTA.glm=glm(tot.abu~cover, family=poisson)

#four parameter logistic regression with poisson error

logip=function(p,lambda,x){

a=p[1]

b=p[2]

c=p[3]

d=p[4]

Riq1 = d+(a/(1+exp((b-cover)/c)))

-sum(dpois(x,lambda=Riq1, log=TRUE))

}

parnames(logip)=c("a","b","c","d")

modTA.log=mle2(minuslog=logip, start= c(a=70,b=25, c=3,d=80), data=list(x=tot.abu))

##null model

modTA.null=glm(tot.abu~1, family=poisson)

#model selection

AICctab(modTA.glm,modTA.log, modTA.null, weights=T, nobs=40)

##null and glm área lmost equiprobable, let us see the points, the models and residuals

#plots:

plot(cover,tot.abu, xlab="Forest cover", ylab="Total Abundance")#original data

abline(h=mean(tot.abu))#null model

points(cover, modTA.glm$fit, type="l", col="dark gray")#glm model

#investigating the glm parameters

coef(modTA.glm)

#a very low value of correlarion coeficient, again.

#null model explins better

#residual analysis

plot(modTA.glm$res, cover)

plot(modTA.null$res, cover)

############

**#Models: generalist birds richness**

#Generalized Linear model

modGR.glm=glm(gen.rich~cover, family=poisson)

#four parameter logistic regression with poisson error

logip=function(p,lambda,x){

a=p[1]

b=p[2]

c=p[3]

d=p[4]

Riq1 = d+(a/(1+exp((cover-b)/c)))#inverse relation

-sum(dpois(x,lambda=Riq1, log=TRUE))

}

parnames(logip)=c("a","b","c","d")

modGR.log=mle2(minuslog=logip, start= c(a=40,b=25, c=3,d=10), data=list(x=gen.rich))

##null model

modGR.null=glm(gen.rich~1, family=poisson)

#model selection

AICctab(modGR.glm,modGR.log, modGR.null,weights=T, nobs=40)

#logistic wons

#investigating

#likelihood profile of parameters

pGR=profile(modGR.log)

#plot

plot(pGR)

#Coefficients:

#a b c d

#14.44908 33.80707 1.38481 13.46904

#plots:

#adjusted points

plot(cover,gen.rich, xlab="Forest cover", ylab="Generalists Richness")#original data

curve (13.46904+(14.44908/(1+exp((x-33.80707)/1.38481))), add=T)

#residual analysis

#generating residuals

resGR.log=gen.rich-(13.46904+(14.44908/(1+exp((cover-33.80707)/1.38481))))

#plot raw residuals against x

plot( cover,resGR.log)

################

**#Models: generalist birds abundance**

#Generalized Linear model

modGA.glm=glm(gen.abu~cover, family=poisson)

#four parameter logistic regression with poisson error

logip=function(p,lambda,x){

a=p[1]

b=p[2]

c=p[3]

d=p[4]

Riq1 = d+(a/(1+exp((cover-b)/c)))#inverse relation

-sum(dpois(x,lambda=Riq1, log=TRUE))

}

parnames(logip)=c("a","b","c","d")

modGA.log=mle2(minuslog=logip, start= c(a=40,b=25, c=3,d=10), data=list(x=gen.abu))

##null model

modGA.null=glm(gen.abu~1, family=poisson)

#model selection

AICctab(modGA.glm,modGA.log, modGA.null,weights=T, nobs=40)

#logistic wons

#investigating

#likelihood profile of parameters

pGA=profile(modGA.log)

#plot

#get coefficients

modGA.log

plot(pGA)

#Coefficients:

#a b c d

#60.704081 32.253299 2.016751 36.678726

#plots:

#adjusted points

plot(cover,gen.abu, xlab="Forest cover", ylab="Generalists Abundace")#original data

curve (36.678726+(60.704081/(1+exp((x-32.253299)/2.016751))), add=T)

#residual analysis

#generating residuals

resGA.log=gen.abu-(36.678726+(60.704081/(1+exp((cover-32.253299)/2.016751))))

#plot raw residuals against x

plot( cover,resGA.log)

###########

**#Models: forest-dependent birds richness**

#Generalized Linear model

modFR.glm=glm(for.rich~cover, family=poisson)

#four parameter logistic regression with poisson error

logip=function(p,lambda,x){

a=p[1]

b=p[2]

c=p[3]

d=p[4]

Riq1 = d+(a/(1+exp((b-cover)/c)))

-sum(dpois(x,lambda=Riq1, log=TRUE))

}

parnames(logip)=c("a","b","c","d")

modFR.log=mle2(minuslog=logip, start= c(a=40,b=25, c=3,d=10), data=list(x=for.rich))

#null model

modFR.null=glm(for.rich~1, family=poisson)

#model selection

AICctab(modFR.glm,modFR.log, modFR.null,weights=T, nobs=40)

#logisticn wons

#investigating

#likelihood profile of parameters

pFR=profile(modFR.log)

#plot

plot(pFR)

#get coefficients from logsitic

modFR.log

#Coefficients:

#a b c d

#21.645268 33.826036 4.750837 12.416747

#plots:

#adjusted points

plot(cover,for.rich, xlab="Forest cover", ylab="Forest-Dependent Richness")#original data

curve (12.416747+(21.645268/(1+exp((33.82603-x)/4.750837))), add=T,lwd=1.5)

#residual analysis

#generating residuals

resFR.log=for.rich-(12.416747+(21.645268/(1+exp((33.82603-cover)/4.750837))))

#plot raw residuals against x

#logistic

plot( cover,resFR.log)

########

**#Models: forest-dependent birds abundance**

#Generalized Linear model

modFA.glm=glm(for.abu~cover, family=poisson)

#four parameter logistic regression with poisson error

logip=function(p,lambda,x){

a=p[1]

b=p[2]

c=p[3]

d=p[4]

Riq1 = d+(a/(1+exp((b-cover)/c)))

-sum(dpois(x,lambda=Riq1, log=TRUE))

}

parnames(logip)=c("a","b","c","d")

modFA.log=mle2(minuslog=logip, start= c(a=80,b=35, c=3,d=40), data=list(x=for.abu))

#null model

modFA.null=glm(for.abu~1, family=poisson)

#model selection

AICctab(modFA.glm,modFA.log, modFA.null,weights=T, nobs=40)

#logistic wons

#investigating

#likelihood profile of parameters

pFA=profile(modFA.log)

#plot

plot(pFA)

#get coefficients from logsitic

modFA.log

#Coefficients:

#a b c d

#67.869384 31.759500 3.140008 45.640603

#plots:

#adjusted points

plot(cover,for.abu, xlab="Forest cover", ylab="Forest-Dependent Abundance")#original data

curve (45.640603+(67.869384/(1+exp((31.759500-x)/4.140008))), col="darkgray", lwd=2,add=T)

#residual analysis

#generating residuals

resFA.log=for.abu-(45.640603+(67.869384/(1+exp((31.759500-cover)/4.140008))))

#plot raw residuals against x

#logistic

plot( cover,resFA.log)

###########

**#Models: frugivorous birds richness**

#Generalized Linear model

modFbR.glm=glm(fru.rich~cover, family=poisson)

#four parameter logistic regression with poisson error

logip=function(p,lambda,x){

a=p[1]

b=p[2]

c=p[3]

d=p[4]

Riq1 = d+(a/(1+exp((b-cover)/c)))

-sum(dpois(x,lambda=Riq1, log=TRUE))

}

parnames(logip)=c("a","b","c","d")

modFbR.log=mle2(minuslog=logip, start= c(a=10,b=30, c=2,d=3), data=list(x=fru.rich))

##null model

modFbR.null=glm(fru.rich~1, family=poisson)

#model selection

AICctab(modFbR.glm,modFbR.log, modFbR.null,weights=T, nobs=40)

#logistic wons

#investigating

#likelihood profile of parameters

pFbR=profile(modFbR.log)

#plot

plot(pFbR)

#get coefficients from logsitic

modFbR.log

#Coefficients:

#a b c d

#6.378173 34.734150 2.264860 3.121281

#plots:

#adjusted points

plot(cover,fru.rich, xlab="Forest cover", ylab="Frugivorous Richness")#original data

curve (3.121281+(6.378173/(1+exp((34.734150-x)/3.121281))), lwd=1.5,add=T)

#residual analysis

#generating residuals

resFbR.log=fru.rich-(7.012601+(12.105213/(1+exp((30.019914-cover)/7.012601))))

#plot raw residuals against x

#logistic

plot( cover,resFbR.log)

##################

**#Models: frugivorous birds abundance**

##Generalized Linear model

modFbA.glm=glm(fru.abu~cover, family=poisson)

#four parameter logistic regression with poisson error

logip=function(p,lambda,x){

a=p[1]

b=p[2]

c=p[3]

d=p[4]

Riq1 = d+(a/(1+exp((b-cover)/c)))

-sum(dpois(x,lambda=Riq1, log=TRUE))

}

parnames(logip)=c("a","b","c","d")

modFbA.log=mle2(minuslog=logip, start= c(a=30,b=35, c=4,d=15), data=list(x=fru.abu))

#null model

modFbA.null=glm(fru.abu~1, family=poisson)

#model selection

AICctab(modFbA.glm,modFbA.log, modFbA.null,weights=T, nobs=40)

#logistic wons

#investigating

#likelihood profile of parameters

pFbA=profile(modFbA.log)

#plot

plot(pFbA)

#get coefficients from logsitic

modFbA.log

#Coefficients:

#a b c d

#25.749904 35.931809 2.629633 12.648185

#plots:

#adjusted points

plot(cover,fru.abu, xlab="Forest cover", ylab="Frugivorous Abundance")#original data

curve (12.648185+(25.749904/(1+exp((35.931809-x)/2.629633))), lwd=1.5,add=T)

#residual analysis

#generating residuals

resFbA.log=fru.abu-(12.648185+(25.749904/(1+exp((35.931809-cover)/2.629633))))

#plot raw residuals against x

#logistic

plot( cover,resFbA.log)

##################

**#Models: insectivores birds richness**

#Generalized Linear model

modIR.glm=glm(ins.rich~cover, family=poisson)

#four parameter logistic regression with poisson error

logip=function(p,lambda,x){

a=p[1]

b=p[2]

c=p[3]

d=p[4]

Riq1 = d+(a/(1+exp((b-cover)/c)))

-sum(dpois(x,lambda=Riq1, log=TRUE))

}

parnames(logip)=c("a","b","c","d")

modIR.log=mle2(minuslog=logip, start= c(a=10,b=30, c=1,d=6), data=list(x=ins.rich))

##null model

modIR.null=glm(ins.rich~1, family=poisson)

#model selection

AICctab(modIR.glm,modIR.log, modIR.null,weights=T, nobs=40)

#logistic wons

#investigating

#likelihood profile of parameters

pIR=profile(modIR.log)

#plot

plot(pIR)

#get coefficients from logsitic

modIR.log

#Coefficients:

#a b c d

#12.105698 30.019599 1.461216 7.012205

#plots:

#adjusted points

plot(cover,ins.rich, xlab="Forest cover", ylab="Insectivores Richness")#original data

curve (7.012205+(12.105698/(1+exp((30.019599-x)/1.461216))), lwd=1.5,add=T)

#residual analysis

#generating residuals

resIR.log=ins.rich-(7.012205+(12.105698/(1+exp((30.019599-cover)/1.461216))))

#plot raw residuals against x

#logistic

plot( cover,resIR.log)

##################

**#Models: insectivororus bird abundance**

#Generalized Linear model

modIA.glm=glm(ins.abu~cover, family=poisson)

#four parameter logistic regression with poisson error

logip=function(p,lambda,x){

a=p[1]

b=p[2]

c=p[3]

d=p[4]

Riq1 = d+(a/(1+exp((b-cover)/c)))

-sum(dpois(x,lambda=Riq1, log=TRUE))

}

parnames(logip)=c("a","b","c","d")

modIA.log=mle2(minuslog=logip, start= c(a=40,b=35, c=1,d=20), data=list(x=ins.abu))

#null model

modIA.null=glm(ins.abu~1, family=poisson)

#model selection

AICctab(modIA.glm,modIA.log, modIA.null,weights=T, nobs=40)

#logistic wons

#investigating

#likelihood profile of parameters

pIA=profile(modIA.log)

#plot

plot(pIA)

#get coefficients from logsitic

modIA.log

#Coefficients:

#a b c d

#56.462531 22.952974 12.643445 5.999742

#plots:

#adjusted points

plot(cover,ins.abu, xlab="Forest cover", ylab="Insectivores Abundance")#original data

curve (5.999742+(56.462531/(1+exp((22.952974-x)/5.999742))), lwd=1.5,add=T)

#residual analysis

#generating residuals

resIA.log=ins.abu-(5.999742+(56.462531/(1+exp((22.952974-cover)/5.999742))))

#plot raw residuals against x

#logistic

plot( cover,resIA.log)

**#####Plot Figure 2**

par(mfrow=c(2,3))

plot(cover, tot.rich, xlim=c(0,100), ylim=c(0,70), xaxp=c(0,100,10), ylab="Richness", xlab=NA, col="black", pch=16,cex.lab=1.3,las="1", main="Total")

abline(h=mean(tot.rich))#null model

plot(cover, for.rich,xlim=c(0,100), ylim=c(0,70), xaxp=c(0,100,10), ylab=NA, xlab=NA, col="black", pch=16,cex.lab=1.3, las="1", main="Forest")

curve (12.416747+(21.645268/(1+exp((33.82603-x)/4.750837))), add=T,col=1)##logistic model

plot(cover, gen.rich,xlim=c(0,100), ylim=c(0,70), xaxp=c(0,100,10), ylab=NA, xlab=NA, col="black", pch=16,cex.lab=1.3, las="1", main="Generalist")

curve (13.46904+(14.44908/(1+exp((x-33.80707)/1.38481))), add=T,col=1)##logistic model

plot(cover,tot.abu,xlim=c(0,100), ylim=c(0,240), xaxp=c(0,100,10), ylab="Abundance", xlab=NA, col="black", pch=16,cex.lab=1.3,las="1")

abline(h=mean(tot.abu))##null model

plot(cover, for.abu, xlim=c(0,100), ylim=c(0,240), xaxp=c(0,100,10), ylab=NA, xlab="Forest Cover (%)", col="black", pch=16,cex.lab=1.3, las="1")

curve (45.640603+(67.869384/(1+exp((31.759500-x)/4.140008))), col=1,add=T)##logistic model

plot(cover, gen.abu,xlim=c(0,100), ylim=c(0,240), xaxp=c(0,100,10), ylab=NA, xlab=NA, col="black", pch=16,cex.lab=1.3, las="1")

curve (36.678726+(60.704081/(1+exp((x-32.253299)/2.016751))), add=T,col=1)##logistic model

**####Plot Figure 3**

par(mfrow=c(2,2))

plot(cover, fru.rich,xlim=c(0,100), ylim=c(0,15), xaxp=c(0,100,10), ylab="Richness", xlab=NA, col="black", pch=16,cex.lab=1.3, las="1", main="Frugivorous")

curve (3.121281+(6.378173/(1+exp((34.734150-x)/3.121281))), col=1,add=T)##logistic model

plot(cover, ins.rich,xlim=c(0,100), ylim=c(0,35), xaxp=c(0,100,10), ylab=NA, xlab=NA, col="black", pch=16,cex.lab=1.3, las="1", main="Insectivorous")

curve (7.012205+(12.105698/(1+exp((30.019599-x)/1.461216))), col=1,add=T)##logistic model

plot(cover, fru.abu,xlim=c(0,100), ylim=c(0,120), xaxp=c(0,100,10), ylab="Abundance", xlab=NA, col="black", pch=16,cex.lab=1.3, las="1")

curve (12.648185+(25.749904/(1+exp((35.931809-x)/2.629633))), col=1,add=T)##logistic model

plot(cover, ins.abu,xlim=c(0,100), ylim=c(0,120), xaxp=c(0,100,10), ylab=NA, xlab="Forest Cover (%)", col="black", pch=16,cex.lab=1.3, las="1")

curve (5.999742+(56.462531/(1+exp((22.952974-x)/5.999742))), col=1,add=T)##logistic model

##############

**#Second - Piecewise model with poisson error for find to threshold value**

modGR.pic=segmented(modGR.glm,seg.Z=~cover,psi=list(cover=c(40)),control=seg.control(display=F))#generalist richness

summary(modGR.pic)

modGA.pic=segmented(modGA.glm,seg.Z=~cover,psi=list(cover=c(40)),control=seg.control(display=F))#generalist abundance

summary(modGA.pic)

modFR.pic=segmented(modFR.glm,seg.Z=~cover,psi=list(cover=c(40)),control=seg.control(display=F))#forest richness

summary(modFR.pic)

modFA.pic=segmented(modFA.glm,seg.Z=~cover,psi=list(cover=c(40)),control=seg.control(display=F))#forest abundance

summary(modFA.pic)

modFbR.pic=segmented(modFbR.glm,seg.Z=~cover,psi=list(cover=c(40)),control=seg.control(display=F))#frugivores richness

summary(modFbR.pic)

modFbA.pic=segmented(modFbA.glm,seg.Z=~cover,psi=list(cover=c(40)),control=seg.control(display=F))#frugivores abundance

summary(modFbA.pic)

modIR.pic=segmented(modIR.glm,seg.Z=~cover,psi=list(cover=c(40)),control=seg.control(display=F))#insectivores richness

summary(modIR.pic)

modIA.pic=segmented(modIA.glm,seg.Z=~cover,psi=list(cover=c(40)),control=seg.control(display=F))#insectivores abundance

summary(modIA.pic)

############

**#Third - Analyze differences in bird composition among landscapes**

**#Forest bird NMDS and ANOSIM**

#loading table of forest bird

for_bird=read.table("c:/data frame/for_bird.txt", header= T, sep="")

#loading table forest cover to forest bird

for_cover=read.table("c:/data frame/for_cover.txt", header= T, sep=""))#site with percentage below 46% (peiecewise value) were categorized as low forest cover and site with cover above 46% were categorozed as high

names(for_cover)

#"Sites" "Cover" "Cat"

#Cat = categories of forest cover (low and high)

plan1=t(for_bird)

plan.pres=plan1

plan.pres[plan.pres>0]=1#transforming the bird records in presence-absence

nmds1=metaMDS(plan.pres,"jaccard", k=2,trymax=20,trace=T)

nmds1$stress

matrix=vegdist(plan.pres)

anosim1=anosim(matrix,for_cover$Cat)

anosim1

#Scores for graphic

scorspec=nmds1$species

scorsitios=nmds1$points

**#Generalist bird NMDS and ANOSIM**

#loading table of generalist bird

gen_bird=read.table("c:/data frame/gen_bird.txt", header= T, sep="")

#loading table forest cover to generalist bird

gen_cover=read.table("c:/data frame/gen_cover.txt", header= T, sep=""))#site with percentage below 50% (peiecewise value) were categorized as low forest cover and site with cover above 50% were categorozed as high

plan2=t(gen_bird)

plan.pres2=plan2

plan.pres2[plan.pres2>0]=1

plan.pres2

nmds2=metaMDS(plan.pres2,"jaccard", k=2,trymax=20,trace=T)

nmds2$stress

matrix2=vegdist(plan.pres2)

anosim2=anosim(matrix2,gen_cover$Cat)

#Scores for graphic

scorspec2=nmds2$species

scorsitios2=nmds2$points

**#Insectivorous bird NMDS and ANOSIM**

#loading table of insectivorous bird

ins_bird=read.table("c:/data frame/ins_bird.txt", header= T, sep="")

#loading table forest cover to insectivorous bird

ins_cover=read.table("c:/data frame/ins_cover.txt", header= T, sep=""))#site with percentage below 44% (peiecewise value) were categorized as low forest cover and site with cover above 44% were categorozed as high

plan3=t(ins_bird)

plan.pres3=plan3

plan.pres3[plan.pres3>0]=1

plan.pres3

nmds3=metaMDS(plan.pres3,"jaccard", k=2,trymax=20,trace=T)

nmds3$stress

matrix3=vegdist(plan.pres3)

anosim3=anosim(matrix3,ins_cover$Cat)

#Scores for graphic

scorspec3=nmds3$species

scorsitios3=nmds3$points

**#Frugivorous bird NMDS and ANOSIM**

#loading table of frugivorous bird

fru_bird=read.table("c:/data frame/fru_bird.txt", header= T, sep="")

#loading table forest cover to frugivorous bird

fru_cover=read.table("c:/data frame/fru_cover.txt", header= T, sep=""))#site with percentage below 46% (peiecewise value) were categorized as low forest cover and site with cover above 46% were categorozed as high

plan4=t(fru_bird)

plan.pres4=plan4

plan.pres4[plan.pres4>0]=1

plan.pres4

nmds4=metaMDS(plan.pres4,"jaccard", k=2,trymax=20,trace=T)

nmds4$stress

matrix4=vegdist(plan.pres4)

anosim4=anosim(matrix4,fru_cover$Cat)

#Scores for graphic

scorspec4=nmds4$species

scorsitios4=nmds4$points

############

**#Plot Figure 4**

par(mfrow=c(2,2))

treat=c(rep("low", 20), rep("high", 20))

plot(scorsitios,type="n",xlim=c(-2,1),ylim=c(-1,1),font.main=1,cex.axis=0.8,cex.lab=0.8,cex.main=0.8,cex=0.6)#forest bird

ordihull(nmds1, groups=treat, draw="lines", col="gray",label=F)

points(scorsitios[1:20,],pch=16,cex=0.9)

points(scorsitios[21:40,],pch=16, cex=0.9)

text(scorsitios,labels=for_cover$Cover,pos=1,cex=0.8)

treat1=c(rep("low", 22), rep("high", 18))

plot(scorsitios2,type="n",xlim=c(-2,1), ylim=c(-1,1),xlab=NA, ylab=NA, font.main=1,cex.axis=0.8,cex.lab=0.8,cex.main=0.8,cex=0.6)#generalist bird

ordihull(nmds2, groups=treat1, draw="lines", col="gray",label=F)

points(scorsitios2[1:22,],pch=16,cex=0.9)

points(scorsitios2[23:40,],pch=16, cex=0.9)

text(scorsitios2,labels=gen_cover$Cover,pos=1,cex=0.8)

treat2=c(rep("low", 20), rep("high", 20))

plot(scorsitios4,type="n",xlim=c(-2,1), ylim=c(-1,1),xlab=NA, ylab=NA, font.main=1,cex.axis=0.8,cex.lab=0.8,cex.main=0.8,cex=0.6)#frugivorous bird

ordihull(nmds4, groups=treat2, draw="lines", col="gray",label=F)

points(scorsitios4[1:20,],pch=16,cex=0.9)

points(scorsitios4[21:40,],pch=16, cex=0.9)

text(scorsitios4,labels=fru_cover$Cover,pos=1,cex=0.8)

treat3=c(rep("low", 20), rep("high", 20))

plot(scorsitios3,type="n",xlim=c(-2,1), ylim=c(-1,1),xlab=NA, ylab=NA, font.main=1,cex.axis=0.8,cex.lab=0.8,cex.main=0.8,cex=0.6)#insectivorous bird

ordihull(nmds3, groups=treat3, draw="lines", col="gray",label=F)

points(scorsitios3[1:20,],pch=16,cex=0.9)

points(scorsitios3[21:40,],pch=16, cex=0.9)

text(scorsitios3,labels=ins_cover$Cover,pos=1,cex=0.8)

**#Fourth - Direct ordination using presence-absence**

#Function to create the graphic

ordination<-function(tabela,gradiente,at,grad,eixoY,eixoX){

tabela<-as.matrix(tabela)

gradiente<-as.matrix(gradiente)

media.pond<-colSums(tabela*gradiente[,1])/colSums(tabela)

sub.orden<-tabela[order(gradiente[,1],decreasing=F),]

sub.orde<-sub.orden[,order(media.pond,decreasing=T)]

dados.pa<-matrix(0,nrow(tabela),ncol(tabela))

dados.pa[tabela>0]<-1

ordenado<-sub.orde[,which(colSums(dados.pa)>0)]

par(mfrow=c(ncol(ordenado)+1,1),mar=c(0,2,0.3,8),oma=c(2.5,1,1,6))

layout(matrix(1:(ncol(ordenado)+1)),heights=c(3,rep(1,ncol(ordenado))))

plot(sort(gradiente[,1]),axes=F,ylab="",mfg=c(21,1),lwd=8,las=2,lend="butt",frame.plot=F,xaxt="n",type="h",col="black",ylim=c(min(gradiente),max(gradiente)))

axis(side=2,at=c(min(gradiente),max(gradiente)),las=2)

mtext(grad,2,outer=T,font=1,line=-24,padj=-34,las=2,cex=0.8)

for(i in 1:ncol(ordenado)){

barplot(ordenado[,i],bty="l",axisnames=F,axes=FALSE,col="black")

mtext(colnames(ordenado)[i],3,line=-1,adj=0,at=at,cex=.55,font=1)

}

mtext(eixoX,1,outer=T,font=1,line=1.2, cex=1)

mtext(eixoY,2,font=1,outer=T,line=-2,cex=1)

}

**#Forest bird-Supplementary material Figure S1**

for.bird=read.csv2("for.bird.csv", header = T, check.names=F)#loading the data containing the species names in the column and sample site in the lines

for.bird[for_bird>0]=1#transforming the bird records in presence-absence

for.bird.plot=ordination(for_bird,cover,50,"Forest Cover (%)","Presence-Absence","Sampling Site")

**#Generalist bird-Supplementary material Figure S2**

gen.bird=read.csv2("gen_bird.csv", header = T, check.names=F)

gen_bird[gen_bird>0]=1

gen_bird.plot=ordination(gen_bird,cover,50,"Forest Cover (%)","Presence-Absence","Sampling Site")

**#Frugivorous bird-Supplementary material Figure S3**

fru_bird=read.csv2("fru_bird.csv", header = T, check.names=F)

fru_bird[fru_bird>0]=1

fru_bird.plot=ordination(fru_bird,cover,50,"Forest Cover (%)","Presence-Absence","Sampling Site")

**#Insectivorous bird-Supplementary material Figure S4**

ins_bird=read.csv2("ins_bird.csv", header = T, check.names=F)

ins_bird[ins_bird>0]=1

ins_bird.plot=ordination(ins_bird,cover,50,"Forest Cover (%)","Presence-Absence","Sampling Site")

**References**

1. Oksanen J, Blanchet FG, Kindt R, Legendre P, Minchin P, et al.. (2013) vegan: community ecology package. R package version 2.0-7. Available: http://CRAN.R-project.org/package=vegan.

2. Venables WN, Ripley BD (2002) Modern Applied Statistics with S. Fourth Edition. New York: Springer.

3. Pinheiro J, Bates D, DebRoy S, Sarkar D, R Core Team (2013) nlme: Linear and Nonlinear Mixed Effects Models. R package version 3.1-113.

4. Bolker BM (2012) bbmle: Tools for general maximum likelihood estimation. R package version 1.0.15. Available: http://CRAN.R-project.org/package=bbmle

5. Muggeo V (2008) Segmented: an R package to fit regression models with broken-line relationships. Rnews 8: 20–25.
